# The role of participatory scenarios in ecological restoration: a systematic map protocol

**DOI:** 10.1186/s13750-022-00276-w

**Published:** 2022-06-22

**Authors:** Eleanor Moore, Pete Howson, Matthew Grainger, Yit Arn Teh, Marion Pfeifer

**Affiliations:** 1 School of Natural and Environmental Sciences, Newcastle University, Newcastle upon Tyne, UK; 2 Department of Social Sciences, Northumbria University, Newcastle upon Tyne, UK; 3 Norwegian Institute for Nature Research, Torgarden, Postbox 5685, 7485 Trondheim, Norway

**Keywords:** Evidence synthesis, Alternative futures, Place-based research, Socioecological systems, Trade-offs, Co-production, Stakeholder engagement

## Abstract

**Background:**

The scale of land degradation worldwide has led the UN to declare the Decade of Ecosystem Restoration and movements such as the Bonn Challenge (https://www.bonnchallenge.org/), have placed ecological restoration on the global policy agenda. Achieving such ambitious policy targets and restoration goals will necessitate complex trade-offs against limited time, competing knowledge, costs, resources, and varying societal preferences among different stakeholders.

Participatory scenarios are a tool to navigate uncertainties surrounding future trajectories and simultaneously incorporate different stakeholder perspectives. They can provide a path to identify collaborative solutions best suited for the local cultures and societies they are tied to. However, there is no systematic understanding of how participatory scenarios are being used in ecological restoration planning to navigate trade-offs in restoration outcomes. We will fill this research gap by mapping the existing evidence from participatory restoration scenarios to answer the primary research question ‘How are outcomes explored in participatory ecological restoration scenarios?’. This will be done through five sub-questions focussing on characteristics of the evidence base, types of study design, how outcomes and trade-offs in those are explored, and an examination of the role of participants in the scenario process and outcome determination.

**Methods:**

This protocol outlines the methods for a systematic map to identify studies that have used participatory scenarios in restoration planning. A comprehensive and reproducible search strategy will be undertaken across bibliographic databases, web-based engines, and targeted searches in organisational online libraries. Searches will be done online in English, but results in all languages will be screened. Search results will go through a two-step screening process of against pre-determined criteria of inclusion and exclusion, for title and abstract and then full-text. Data will be extracted from eligible studies using a standardised data extraction spreadsheet where details on study characteristics, design and outcomes will be recorded. A searchable database of studies and mapping outcomes will be available upon completion of the work. The aim is to inform how scenarios can be better used as a decision-making tool to increase stakeholder participation and account for trade-offs in restoration outcomes across social, ecological, and economic dimensions.

**Supplementary Information:**

The online version contains supplementary material available at 10.1186/s13750-022-00276-w.

## Background

The scale of land degradation worldwide has led the UN to declare 2021–2030 the Decade of Ecosystem Restoration and targets such as the Bonn Challenge that aims to restore 350 million hectares by 2030 have placed ecological restoration on the global policy agenda [1, 2]. Ecological restoration is an important tool for managing and improving highly degraded or altered environments [3]. We define ecological restoration as “the process of assisting the recovery of a degraded, damaged, or destroyed ecosystem to reflect values regarded as inherent in the ecosystem and to provide goods and services that people value” [4]. This definition incorporates the social aspect that drives restoration planning and implementation by including the motivating rationale [4].

Currently, under the Bonn Challenge, 210 million hectares have been pledged across 71 countries, although the extent of success is unclear and will not be evident for some time [2, 5]. Achieving such ambitious policy targets and restoration goals will necessitate complex trade-offs against limited time, competing knowledge, costs, resources, and varying societal preferences among different stakeholders. There is also surmounting evidence of the value of incorporating traditional or local ecological knowledge in restoration projects [6, 7]. In this light, we need strategic planning that takes a holistic approach to the social, ecological, and economic complexities while also including and respecting different forms of knowledge [8].

### Participatory scenarios

Scenarios are representations or storylines of possible futures [9]. They are useful in restoration planning, often a short-term process that aims to achieve long-term outcomes. Scenarios can be applied to restoration planning for a variety of objectives, including to explore uncertainties or understand the effect of a specific management intervention on restoration objectives, or to understand the effect of different management interventions on specific desired outcomes [10]. As a decision-making tool, the outcomes can be used to prioritise (in space and time) decisions and resources and to reduce costs [11]. The integration of participatory methods in scenario planning provides a path to identify collaborative solutions best suited for the local cultures and societies they are tied to [12]. It is now widely considered in the literature and international standards that active engagement with stakeholders will underpin long-term restoration success [11, 12]. Stakeholder participation is “a process where individuals, groups and organisations choose to take a role in making decisions that affect them” [13]. There is an emerging consensus that the probability of multiple positive outcomes for biodiversity and livelihoods in forest systems increases with participation in decision-making [14].

Participatory scenarios are a tool to navigate uncertainties surrounding future trajectories and simultaneously incorporate different stakeholders [15, 16]. For instance, Palacios-Agundez et al. [17] downscaled the Millennium Ecosystem Assessment global scenarios with stakeholders in Basque Country, Spain. Participants were able to relate the global scenarios to local drivers of change, and suggest management actions towards achieving their desirable scenario framed by the local culture and context [17]. The authors reported participants learned to see and understand different perspectives and collaborated on proposing feasible management responses. This holistic approach to the future that seeks to manage uncertainties with stakeholders and compare different possible outcomes of decisions is what separates the method from others such as Theories of Change [18] and Participatory Rural Appraisal [19].

Despite extensive reports of success in participatory methods for environmental management, there is also evidence of them failing to meet their objectives [20, 21]. Assessment suggests that for example, a lack of participant diversity and representativeness may lead to outcomes not reflecting the multitude of viewpoints within a community, undermining a key motivation for using participatory methods [21]. Guidance to implement participatory scenarios in restoration planning has been developed [9, 10]. Recent advice, drawing from the six best practice principles for scenarios in restoration planning, Metzger et al., [10] concludes that stakeholders should participate through the whole process; from method planning to creation and review of scenarios.

### Trade-offs in achieving desired outcomes

Ecological restoration projects are often no longer about solely achieving ecological success. Instead they are positioned alongside narratives of ‘win–win’ and win-lose, aiming to investigate restoration legacies for improved social outcomes such as enhanced livelihoods and climate change mitigation [1, 25, 26]. Previous research has noted that ecological indicators have often focussed on structure or composition as surrogates for ecosystem functioning [27] but called for greater focus on social in addition to ecological outcomes [28]. Yet, traditionally ecological restoration has predominantly focused ecological outcomes and this is insufficient to understand the array of impacts on the socio-ecological system targeted by restoration interventions [27–29].

Stakeholder engagement and scenarios have been suggested as a key tool to analyse trade-offs in outcomes across time, space, and stakeholders [10, 30, 31]. Participatory scenario methods facilitate discussions around outcomes and how stakeholders may respond to interventions allowing for social and economic dimensions to also be captured [32]. They also allow for the integration of multiple disciplines and methods, for instance, Bremer et al., [15] quantified ecological and economic outcomes in scenarios of grassland restoration alongside qualitative evaluation of cultural ones.

Determining and balancing outcomes of restoration objectives can be challenging because values differ between stakeholders, who often have diverse knowledge systems and expectations [33, 34]. Using scenarios allows us to investigate alternative futures and evaluate the inherent trade-offs that may need to be made, looking to reduce uncertainty [35]. Besides, trade-offs tend to feature heavily in participants discussions, even when they are not explicitly addressed [32]. For example, when planning restoration of native grassland in central France, sheep farmers’ primary objective was maintaining sheep production while conservationists were concerned with the preservation of local biodiversity [32]. Furthermore, even if restoration objectives are agreed upon, the interventions suggested to achieve them can be vastly different between stakeholders. In the restoration of ponderosa pine forests to reduce fire risk, some participants recommended no treatment while others recommended extreme thinning at the same location in the landscape [36]. Despite a push towards participatory methods, a review by Acosta et al. [22] into scenarios for restoration planning found only 11% of publications adopted a participatory approach. There is currently little known on the extent of participation by stakeholders in participatory scenarios [22], and this will vary depending on study context, time, and resources available. Here we aim to take a broad definition of participation that stakeholders must play a role in decision-making at any stage in the scenario process, and this may not necessarily be throughout the whole process. This role in decision making can take many different forms from one-way engagement such as consultation, to co-production in which there is two-way knowledge exchange and production [23, 24].

There currently is no systematic understanding of how participatory scenarios are being used in ecological restoration, including the geographic and spatial scope through which they are applied, the types of restoration projects they are being used for and the restoration outcomes they are addressing. Moreover, despite calls for the need to include of a broad range of outcomes and indicators there is insufficient evidence this is the case [37]. We will fill this research gap by mapping the existing evidence from participatory restoration scenarios to examine how restoration outcomes are explored using participatory scenarios and how participants are involved in the scenario process. We will determine what outcomes and trade-offs are being examined using participatory scenarios, and where there are knowledge gaps to improve further research.

### Stakeholder involvement

This systematic map is being led by a team at Newcastle University and Northumbria University. The authors all specialise within the fields of ecological restoration across both ecological and social sciences. The main aims were formulated between the review team and then it was sent to five external experts in environmental restoration to review. These experts were purposefully selected from the networks of the review team because they have experience in different regions and topic areas within restoration. They provided a broader understanding of the topic, knowledge gaps and contributed to the list of synonyms for the search string. Through publishing the protocol with Environmental Evidence, we have adhered to their review standards and taken advantage of being able to undergo a peer review process for the protocol and gain valuable feedback to improve the final systematic map. When undertaking the review, we will do a call for submissions through organisational networks, an open call through social media and review team networks. Once the map is finished, we plan to do a one page summary with infographics for academics and practitioners to disseminate results.

### Research objectives

The aim of this systematic map is to inform how scenarios can be better used as a decision-making tool to increase stakeholder participation and account for trade-offs in restoration outcomes across social, ecological, and economic dimensions. The research question was designed using the SPIDER framework because of the qualitative and mixed method nature of the literature base [38] (Table 2). This led to the following overarching research question:

How are outcomes explored in participatory ecological restoration scenarios?

The main research question will be answered through the following sub-questions:

- What are the characteristics of the current evidence base – location, scale, design, restoration intervention type?
- What types of study designs are used for participatory scenarios in restoration planning?
- What types of outcomes are explored using participatory scenarios?
- How are trade-offs in outcomes explored in participatory scenarios?
- What is the role of participants in the scenario process and outcome determination?

## Methods

### Search strategy

A benchmark list of eight articles was created that captured a range of relevant studies for the review through scoping searches and bibliographies of relevant papers. Search terms were collated through keywords extracted from the benchmark papers, consultation with academic experts, a librarian, and a thesaurus [see Additional file 1]. Search terms were based on the key components of the question. The search string was developed using the Web of Science Core Collection and Boolean operators. Themes were combined using “AND”, while synonyms within themes were combined using “OR”. Combinations of different synonyms were then applied to searches and tested against a list of benchmark articles [see Additional file 2]. The final search string was the minimum number of terms to gain both high sensitivity but low specificity. The search string and all searches will be conducted in English but results in all languages will be screened.

Final search string based on of the research question:

Scenario: Scenario* OR forecast* OR backcast* OR futur* OR trajector*

AND

Participatory: participat* OR collabor* OR co-product* OR collectiv* OR stakehold* OR engag*

AND

Ecological: ecolog* OR environment* or ecosystem*

AND

Restoration: restor* OR reveg* OR regener* OR reforest* OR afforest* OR remediat* OR rehabilitat* OR rewild* OR re-wild* OR "conservation translocat*".

*is a wild card and includes any characters on the end of the word. For example, participat* may include participation, participatory, participative.

### Bibliographic database searches

Bibliographic database searches will follow the logic of the search string described above [see Additional file 1 for search string details for each database]. The following bibliographic databases will be searched:

1. Web of Science by Thomson-Reuters (All collections): A multi-disciplinary database of peer-reviewed scientific literature, books, book chapters and conference proceedings [39].
2. Scopus by Elsevier: A multi-disciplinary database of peer-reviewed scientific literature, books, book chapters and conference proceedings [40].
3. Cab abstracts by CAB international: covers research in the fields of agriculture and land use [41].
4. ProQuest (Natural Science Collection and Social Science Collection): A full range of natural science and social science databases [42, 43].
5. Lens.org [44]

### Web-based search

An internet search will be performed using Google Scholar. Due to search capability limitations, a simplified modified search string will be used: “scenario” AND “participatory” OR “collaborative” AND “restoration” OR “regeneration” OR “reforestation” AND “ecological” OR “ecosystem”. The first 500 terms will be screened.

### Specialist search for grey literature

Grey literature targeted searches were determined through two avenues. Firstly, through discussion with six academic experts in restoration or participatory research identified through the reviewers' academic network. Secondly, through scoping searches for organisations linked to the Bonn Challenge or UN Decade of Restoration. Due to limitations in search capabilities and to ensure a broad search in these databases, only the term ‘scenario’ will be searched for and then all results screened. A full list of the organisations is found in Table 1.

**Table 1:** A list of the organisations and accompanying URLs that will be searched

Organisation	Link as of (19/11/2021)
International Union for Conservation of Nature (IUCN)	https://portals.iucn.org/library/
Food and Agriculture Organisation of the United Nations (FAO)	http://www.fao.org/scripts/catweb/frees.htm
Society for Ecological Restoration (SER)	https://www.ser-rrc.org/resource-database/
Global Landscapes Forum (GLF)	https://www.globallandscapesforum.org/#
Landscapes for People, Food and Nature	http://peoplefoodandnature.org/
World Resources Institute	https://www.wri.org/
Stockholm Resilience Centre	https://www.stockholmresilience.org/
UN-REDD	https://www.un-redd.org/
WWF	https://wwf.panda.org/
Tropenbos International	https://tropenbos.org
Ecoagriculture Partners	https://ecoagriculture.org/
International Tropical Timber Organisation	https://www.itto.int/
World Agroforestry (ICRAF)	https://www.worldagroforestry.org/
Center for International Forestry Research (CIFOR)	https://www.cifor.org/
Consultative Group on International Agricultural Research (CGIAR)	https://www.cgiar.org/
European Forest Institute	https://efi.int/
Rainforest Alliance	https://www.rainforest-alliance.org/

### Targeted searching

The bibliographies and citing articles of other relevant evidence syntheses or publications on the use of participatory scenarios in natural resource management will be screened for relevant literature. This will include already discovered literature [10, 22, 32], and any found during the screening process. Acosta et al., [22] will also be contacted for the list of included studies of their evidence synthesis to be screened.

### Other literature searches

We will make use of social media channels and email list serves to inform the ecological restoration community of this review and request submissions of relevant literature (both scientific and grey). A targeted call for evidence will be placed within the International Union for Conservation of Nature, Science for Nature and People network and the Global Landscapes Forum. All submitted publications will be screened for eligibility according to the methods described below.

We will also perform forward and backward citation chasing using the citationchaser package [45] on all publications that pass full-text screening. Duplicates will be removed and the remaining publications will be screened for eligibility.

## Article screening and study eligibility

### Pilot testing

The eligibility criteria were pilot-tested by three reviewers. Firstly, reviewers screened the titles and abstracts of the test papers. Screening decisions were compared, and any inconsistencies were discussed, and the criteria subsequently adjusted. Then, the process was repeated for full-text versions of the test list. Once the eligibility criteria were set, the data extraction codebook was also pilot tested by three reviewers on the test list. Any inconstancies were discussed, and the codebook was adjusted accordingly.

### Study eligibility

Each publication will be screened according to the eligibility criteria in Table 2, all publications must also be available in an online format. A list of excluded articles with the reason for exclusion will be included in full text.

**Table 2:** Description of each question components using the SPIDER framework and the accompanying inclusion and exclusion criteria for publication screening

SPIDER framework	Question component	Criteria
Sample	Participants	Inclusion: The research has some form of participation with stakeholders as defined “A process where individuals, groups and organisations choose to take an active role in making decisions that affect them” [12]. Participation can be at any stage in the scenario construction process, for example, input into scenarios that are then used in modelling, data collection with participants or feedback from participants on scenario outputs Stakeholders include everyone directly or indirectly affected by the restoration planning or future scenarios discussed, but they must be outside the investigation team
Phenomenon	Ecological restoration	Inclusion: The publication must address any form of ecological restoration as per the definition “Ecological restoration is the process of assisting the recovery of a degraded, damaged, or destroyed ecosystem to reflect values regarded as inherent in the ecosystem and to provide goods and services that people value” [4] Types of restoration may include, but are not limited to: landscape, species, ecosystem, ecosystem service, native species, invasive species removal, habitat, water catchment, coastal, marine Ecological restoration may be addressed through either of the following criteria: 1.The main goal of scenario building is explicitly for ecological restoration 2.The main goal of the scenario building is not explicitly stated as restoration within the publication however it must be identifiable to coders. For instance, if the study area or associated ecological functions are described as degraded and the scenarios are addressing the future of these components 3.Ecological restoration is not the main aim of building scenarios, but features as a possibility from at least one scenario. For example, one scenario may be ecological restoration while another may be conversion to an alternative land use
Design	Future scenarios	Inclusion: Publication must build or evaluate as least one ‘scenario’ per the definition “Plausible representations of possible futures for one or more components of a system, or as alternative policy or management options intended to alter the future state of these components” (9)
Evaluation	Outcomes	Inclusion: The systematic map will be displaying what and how outcomes are explored within eligible study types. All types of outcomes are eligible but they must be explored in a future scenario
Research type	Qualitative, quantitative, mixed method	Inclusion: Methods used may be qualitative, quantitative or a mixed methods approach

### Screening process

A two-stage screening will take place: title and abstract screening and then full-text. Title and abstract screening will be conducted using the web programme Rayyan [46]. At each stage, inclusion will be determined through the eligibility criteria (Table 2). Suppose the reviewer is unsure or there is not enough information to determine eligibility at the title and abstract stage. In that case, the study will be included in the full-text stage. Doubts of inclusion at the full-text stage will be discussed and determined by the review team, the majority decision will be taken forward. A complete list of excluded studies alongside the reasoning will be included upon publishing the review as required by the RepOrting standards for systematic Evidence Syntheses standards (ROSES) [47] [Additional file 3].

The screening process will be conducted and be predominantly performed by the primary reviewer. At each stage, a random 30% subset of articles will be screened by a minimum of two other reviewers. Randolph’s free-marginal Kappa coefficient will be calculated to test for consistency [48]. This measures the agreement between reviewers corrected for how often this may be due to chance. Any disagreements between reviewers will be discussed, and the decision to include or exclude the article made together.

The reviewers do not expect to be authors on any of the publications included in the final review. If this does occur, then it will be declared, and detailed reasoning for each decision will be reported.

### Data coding and extraction strategy

Studies that have met the eligibility criteria at full-text screening will undergo data coding and extraction. Each publication will be assessed using a codebook to capture the relevant data. The data extraction template consists of pre-defined fixed answers and open questions [Additional file 4]. A minimum of one other reviewer will code a random 20% of articles. Any differences will be discussed and adjusted according to what is agreed between the reviewers. If critical information is missing or unclear, the lead author of the study will be contacted and asked to provide the information. The extracted data records will be made available as additional files upon publishing.

The data to be extracted will be grouped into the following categories and subcategories based on the themes of the research questions:

1. Data coding of study characteristics
  - Bibliographic information
  - Study context
  - Restoration context
2. Scenarios
  - Methods
  - Outcomes
  - Trade-offs in outcomes
  - Analysis and dissemination
3. Participation information
  - Participant selection
  - Participant engagement in the process

For each outcome explicitly addressed, we will classify the outcomes explored in each study into whether they are social, ecological, or economic. The indicators used for each outcome will be listed and ecological outcomes and indicators will be further categorised into compositional, structural, or functional restoration attributes [28, 49]. Compositional indicators refer to the identity and variety of elements of the system such a species richness and diversity [50], while structural indicators are measuring the physical organisation of the systems like tree height and diameter [51]. Functional indicators identify the ecological processes, these often relate to soils such as chemical composition or bioindicator assemblages [49, 51]. We will extract the reasoning given for choosing each outcome, and how it was analysed (qualitatively or quantitatively and methods).

Each study will be documented as to whether analysis of trade-offs in outcomes is explicit or implicit and whether they are explored across space, time, and stakeholders. Each outcome will also be classified as to whether it is analysed as a trade-off and if it is, whether the individual outcome was always positive, always negative, or dependent on the scenario. We will also extract the method stage that trade-offs are analysed and how this occurs in the publication.

To understand participation, we will first look at how participants were chosen, how many were included and which stakeholders were involved. Stakeholders will be categorised, eg. Academics, NGO, government, and we will record if participants are involved in development of scenario objectives, design of methods, scenario creation, analysis of outcomes and trade-offs and dissemination of results. For each participatory stage, we will record the method of participation used.

### Study mapping and presentation

To identify characteristics of the evidence base and explore patterns in the data, the publication years, temporal and spatial scale of the scenarios, and restoration type will be visualised alongside a narrative synthesis. A geographic map will display the location of studies. A narrative synthesis will be used to summarise the methods being used and describe trends between methodologies and study characteristics. This will be accompanied by a visual representation in the form of a decision tree will display the different passages from method planning to outputs.

Patterns will be reported in which categories of outcomes are analysed and trends within each group to identify underrepresented outcomes. The reasons for choosing outcomes will be narratively synthesised alongside how they were analysed within the scenarios. We will also report on trends of which outcomes were usually reported to have a positive trade-off and which a negative, and if trade-offs are explored across different scales. A spider diagram based on frequency of studies will show the different types of trade-offs being analysed and identify knowledge gaps and clusters.

We will use narrative synthesis to describe how participants are chosen and how many are included. Stakeholder categories will be visualised using a graph. A visualisation using a heat map to display a gradient of higher to lower participation in the different stages of the scenario process will be created based on frequency of studies to highlight underrepresented stages.

Results be presented as a publication and a machine-readable and human-readable format that ensures a searchable list of both included studies and the extracted meta-data. Machine-readable formatting will follow the guidelines of Haddaway et al. [52] to ensure design allows for translation, has appropriate naming conversions and is clear to use. This way, searches can be done against criteria such as geographic location or restoration type.

## Supplementary Information

**Additional file 1. **SM_1_Search-strategy. Search Strategy development. Document containing the test list of benchmark articles, key word development and final search string by database.

**Additional file 2. **SM_2_Search_string_building. Search string development to develop the final search string in Web of Science.

**Additional file 3. **SM_3_ROSES for Systematic Map Protocols. ROSES checklist.

**Additional file 4.** SM_4_Data_extraction. The data extraction code book.

## Data Availability

Data sharing is not applicable to this article as no datasets were generated or analysed during the current study.
