# Supplementary material for: The role of participatory scenarios in ecological restoration: a systematic map protocol
Source: Environ Evid. 2022 Jun 22;11:23. doi: 10.1186/s13750-022-00276-w (PMC11378783; doi:10.1186/s13750-022-00276-w)
Supplement: Supplementary file 3 — Additional file 3. SM_3_ROSES for Systematic Map Protocols. ROSES checklist. [file 13750_2022_276_MOESM3_ESM.pdf]

Section / sub-secti	Topic	Description	Further explanation	Checklist/Meta-da	Author response	Comments
Title	Title	The title must indicate th	The title should normally	Meta-data	The role of participatory scenarios in ecological restoration: A systematic map protocol	
Type of review	Type of review	Select one of the followir	See CEE Guidance on syst	Meta-data	systematic map	
Authors contacts	Authors contacts	The full names, institutional addresses, and email a		Checklist	Yes	
Abstract	Structured summary	Abstract must not exceed 350 words and must incl		Checklist	Yes	
Background	Background	Describe the rationale fo	A theory of change and/c	Checklist	Yes	
Stakeholder engage	Stakeholder engagement	The planned/actual role of stakeholders throughou		Checklist	Yes	
Objective of the rev	Objective	Describe the primary que	The primary question is t	Checklist	Yes	
	Definitions of the question	Break down and summar	For other question types	Meta-data	Yes	SPIDER framework used.
Methods						
Searches	Search strategy		Details regarding search	Checklist	Yes	
	Search string	Provide Boolean-style full search string and state tl		Meta-data	(((TS=(restor* OR reveg* OR regener* OR reforest* OR afforest* OR remediat* OR rehabilitat* Or rewild* OR re-wild* OR "conservation transloc*") AND TS=(participat* OR collabor* OR co-product* OR collectiv* OR stakehold* OR engag*) AND TS=(ecolog* OR environment* OR ecosystem*) AND TS=(Scenario* OR forecast* OR backcast* OR futur* OR trajector*))))	Web of science format
	Languages – bibliographic d	List languages to be used in bibliographic database		Meta-data	All	
	Languages – grey literature	List languages to be used in organizational website		Meta-data	All	
	Bibliographic databases	Provide the number of bibliographic databases to b		Meta-data	5	
	Web – based search engine	Provide the number of web – based search engine:		Meta-data	1	
	Organisational websites	Provide the number of organisational websites to b		Meta-data	17	
	Estimating the comprehens	Describe the process by which the comprehensive		Checklist	Yes	
	Search update	Describe any plans to up	Optional. A search updat	Checklist	n/a	
Article screening ar	Screening strategy	Describe the methodology for screening articles/st		Checklist	Yes	
	Consistency checking	Describe clearly the process for checking consisten		Checklist	Yes	
	Inclusion criteria	Describe the inclusion criteria used to assess releva		Checklist	Yes	
	Reasons for exclusion	State that you will provide a list of articles exclude		Checklist	Yes	
Critical appraisal	Critical appraisal strategy	Describe here the metho	Optional	Checklist	n/a	
	Critical appraisal used in sy	Describe how the inform	Optional	Checklist	n/a	
	Consistency checking	Describe how repeatabili	Optional	Checklist	n/a	
Data extraction	Meta-data extraction and c	Describe the method for meta-data extraction and		Checklist	Yes	
Data synthesis and	Narrative synthesis strategy	Describe methods to be u	Vote-counting (tallying o	Checklist	Yes	
	Knowledge gap and cluster	Describe the methods to be used to identify and/o		Checklist	Yes	
	Demonstrating procedural i	Describe the role of syste	Reviewers who have auth	Checklist	Yes	
Declarations	Competing interests	Describe of any financial or non-financial competir		Checklist	Yes	
